# Helminthic Infections and Vaccine Efficacy in Cattle: Implications for Disease Control and Sustainable Livestock Production

**DOI:** 10.3390/vetsci13010018

**Published:** 2025-12-24

**Authors:** Teresa Freire, Alejandra V. Capozzo

**Affiliations:** 1 Laboratorio de Inmunomodulación y Vacunas, Unidad Académica de Inmunobiología, Facultad de Medicina, Universidad de la República, Montevideo 11800, Uruguay; 2 Centro de Altos Estudios en Ciencias Humanas y de la Salud, CONICET-Universidad Abierta Interamericana, Montes de Oca 745, Buenos Aires 1270, Argentina; alejandravictoria.capozzo@uai.edu.ar

**Keywords:** helminth infection, *Fasciola hepatica*, *Ostertagia ostertagi*, cattle vaccination, immune modulation, vaccine efficacy, diagnostic-based parasite control, integrated health management, livestock immunology, sustainable production

## Abstract

Vaccination remains a cornerstone of livestock disease control, yet its effectiveness under field conditions is often compromised by concurrent infections, particularly parasitic helminths. This review explores how infections shape vaccine-induced immunity in cattle, emphasizing the immunoregulatory mechanisms by which helminths interfere with protective responses. Chronic infections with *Fasciola hepatica* and *Ostertagia ostertagi* induce Th2-biased and regulatory immune environments that suppress antigen presentation, cytokine production, and memory formation and maintenance, leading to reduced vaccine efficacy. Evidence from experimental and field studies is scarce and constitutes a gap in our knowledge on how vaccines work in the field. Available data indicate that infection timing, intensity, and chronicity critically determine the extent of vaccine interference. The review highlights diagnostic approaches that can support targeted deworming before vaccination and proposes integrated management strategies combining parasite control, immunization, and nutritional optimization. Such approaches can mitigate helminth-driven immune suppression, enhance herd protection, and reduce dependence on anthelmintics. However, the impact of helminth infections on vaccine efficacy in cattle should be further assessed in the field. Understanding parasite–vaccine interactions is essential to refine vaccination programs, guide the development of next-generation vaccines, and promote sustainable livestock health in parasite-endemic areas.

## Simple Summary

Livestock vaccines are essential for animal health and food production, yet their effectiveness can be reduced by common infections that alter how animals respond to immunization. Among these, parasitic worms such as *Fasciola hepatica* and *Ostertagia ostertagi* play a major role by changing the balance of the immune system, making it less able to develop strong and lasting protection after vaccination. This review describes how these parasites influence immune function in cattle and why infected animals often show weaker or delayed responses to vaccines. It also discusses practical strategies to reduce this problem, such as combining vaccination with timely parasite control, better diagnostic tools, and improved farm management. Understanding the interaction between infection and vaccination is vital to design more effective disease prevention programs and to improve animal welfare, productivity, and sustainability. These insights are especially important for farmers and veterinarians working in regions where parasitic infections are common and may help guide policies that enhance the global efficiency of livestock vaccination.

## 1. Introduction

Animal production represents a cornerstone of global food security, rural livelihoods, and human health. Cattle, swine, poultry, and small ruminants provide high-quality protein in the form of meat, milk, and eggs, all essential for a balanced diet. Beyond nutrition, livestock contributes to economic stability, cultural heritage, and international trade. The quality, safety, and nutritional value of animal-derived products are intimately linked to the health and welfare of the animals themselves, highlighting the necessity of sustainable and well-managed production systems [1]. Ensuring product quality requires not only efficient husbandry but also comprehensive disease prevention strategies, which minimize zoonotic risk, reduce antimicrobial use, and prevent economic losses.

Ensuring animal health is fundamental for both productivity and food safety. Preventive measures, including biosecurity, proper nutrition, and vaccination, play a critical role in minimizing disease incidence. Among these, vaccination remains the most effective, cost-efficient, and environmentally sustainable tool for preventing and controlling infectious diseases. Vaccination reduces morbidity and mortality, improves animal welfare, enhances productivity, and decreases the need for antimicrobials, thereby contributing to global efforts against antimicrobial resistance [2]. Even vaccines against viruses contribute to antimicrobial resistance by disabling the immune suppression usually induced by viral infections that predispose individuals to bacterial diseases.

Most veterinary vaccines are developed under Good Manufacturing Practices (GMP) and tested in laboratory animals. Some regulatory bodies also require that vaccines be tested in the targeted species, and this is performed under controlled experimental conditions using healthy, young animals, which may not represent the immunological diversity and environmental challenges present in real production settings. Post-licensure (Phase IV-equivalent) field evaluations, which are standard practice in human vaccinology, are seldom implemented in livestock, limiting our understanding of how vaccines perform in populations affected by concurrent infections, nutritional stress, or climatic and management stressors [3]. This gap constrains the ability to design evidence-based vaccination programs tailored to diverse epidemiological and production contexts.

This review examines how infections influence vaccination efficacy in cattle, focusing on the immunological mechanisms by which helminth parasitic pathogens modulate vaccine responses. It discusses the interactions between infection-induced immune regulation and vaccination outcomes, emphasizing examples where co-infections reduce, delay, or skew vaccine-induced protection. By integrating data from field studies and experimental models, this review aims to identify knowledge gaps that limit vaccine predictability and to propose priorities for improving vaccine assessment in real-world field conditions. Understanding these dynamics is essential not only for optimizing disease control but also for advancing the sustainability and resilience of livestock production systems in a changing global health landscape.

## 2. Veterinary Vaccines

Depending on the epidemiological situation and national regulations, vaccines may be compulsory, particularly those targeting transboundary or zoonotic diseases, or optional, aimed at improving herd health and productivity within specific management systems. Yet, vaccine efficacy is not absolute, as it depends on multiple factors. Understanding how these factors interact is fundamental to optimising vaccination outcomes and ensuring sustainable, high-quality animal production.

Veterinary vaccines encompass a broad spectrum of technological platforms tailored to the specific needs of various species and pathogens. Traditional vaccines include live attenuated preparations, such as those against bovine viral diarrhea virus (BVDV), porcine reproductive and respiratory syndrome virus (PRRSV), and Newcastle disease virus, as well as inactivated formulations, widely used for foot-and-mouth disease (FMD), classical swine fever (CSF), and avian influenza. Advances in biotechnology have introduced recombinant vector vaccines, such as adenovirus- or vaccinia-vectored PRRSV and recombinant bovine herpesvirus-1 (BHV-1) glycoprotein D vaccines, and subunit vaccines, exemplified by recombinant E2 glycoprotein for CSF and hemagglutinin-based vaccines for avian influenza. Emerging platforms, including mRNA and nanoparticle-based vaccines, hold promise for rapid, scalable, and precisely targeted immunisation, although their application in veterinary medicine remains largely experimental.

Effective vaccination depends on the induction of a robust, durable, and appropriately directed immune response, which is not always fully characterized. The absence of suitable correlates of protection is also a gap that compromises post-vaccination monitoring in field conditions. Vaccine performance is influenced by the interplay between host, pathogen, and management factors. Host-related variables, such as genetics, age, immune status, and nutritional condition, modulate the magnitude and quality of the response [4]. Pathogen-related elements, including strain variation and antigenic diversity, affect the breadth of protection. Management factors, such as stress, co-infections, and failures in cold-chain maintenance or administration timing, can further compromise immune priming [5]. Concurrent infections and environmental stressors may divert immune resources, reduce antigen presentation, or alter cytokine signaling networks, ultimately diminishing vaccine efficacy.

A comprehensive understanding of these determinants is essential to design immunization strategies that elicit effective and long-lasting protection under diverse field conditions. Integrating this knowledge into vaccine development, regulatory assessment, and field evaluation will contribute to more predictable vaccine performance and strengthen the sustainability and resilience of livestock production systems.

## 3. Vaccine Immunology

Vaccines elicit immune responses that can be categorized into T-cell-dependent (TD) and T-cell-independent (TI) pathways. TD responses, typically induced by protein antigens, require antigen processing and presentation by antigen-presenting cells (APCs), followed by activation of helper T cells (CD4^+^) and subsequent B cell activation and class switching. This pathway leads to the generation of long-lived memory B cells and plasma cells, providing robust and durable immunity. In contrast, TI responses, often triggered by polysaccharide antigens, can activate B cells directly without T cell help, resulting in rapid antibody production. However, these responses are generally short-lived and lack the formation of immunological memory. Studies in cattle have demonstrated that TI antigens can induce IgM responses but fail to generate lasting plasma cell populations, highlighting the limitations of TI immunity in veterinary species [6].

Among the factors that can influence vaccine-induced immunity in cattle, maternal antibodies are of major importance. They provide early passive protection but may also interfere with vaccine antigen recognition by neutralizing epitopes before they are processed by the neonate’s immune system. This interference can delay the onset of active immunity and reduce vaccine efficacy, as observed in young animals vaccinated in the presence of high maternal antibody titres. Indeed, studies have shown that maternally derived neutralizing antibodies can impair the efficacy of vaccines against pathogens like FMD virus or BVDV. Interestingly, vaccine-induced antibody responses, but not T cell responses, were impaired by the presence of maternal antibodies [7,8]. Notably, high levels of maternal antibodies have been shown to reduce neutralizing antibody seroconversion by 40–70% in calves vaccinated against FMDV, despite detectable T-cell responses. Concurrent infections can further modulate cytokine networks or alter APC function, diverting immune responses away from the vaccine antigen and leading to incomplete or short-lived protection [9]. Understanding how these variables shape both humoral and cellular responses is essential for optimizing vaccination schedules and improving disease control in cattle populations.

## 4. Impact of Infections on Innate and Adaptive Immunity

Infections can significantly modulate both innate and adaptive immune responses. Pathogens can alter the function of APCs, such as dendritic cells and macrophages, affecting their ability to process and present antigens effectively. This impairment can lead to suboptimal activation of T and B lymphocytes, disrupting the initiation of adaptive immune responses. Additionally, infections often result in dysregulated cytokine production, which can skew immune responses and contribute to immunopathology. Chronic infections can induce a state of immune exhaustion marked by sustained antigen stimulation, overproduction of inflammatory mediators, and the upregulation of inhibitory receptors on T cells. These changes result in diminished cytokine production, reduced proliferation, and impaired effector function. In this sense, a systematic review by Howell et al. found that liver fluke infections in cattle are often associated with reduced cellular immune responses to *Mycobacterium bovis* diagnostic tests, likely due to Th2/Treg skewing and anti-inflammatory cytokines induced by helminths [10]. Although effects were modest, suppression of Th1-associated assays such as the skin test and interferon-γ release assays was consistently reported across observational and experimental studies.

**Figure 1:**
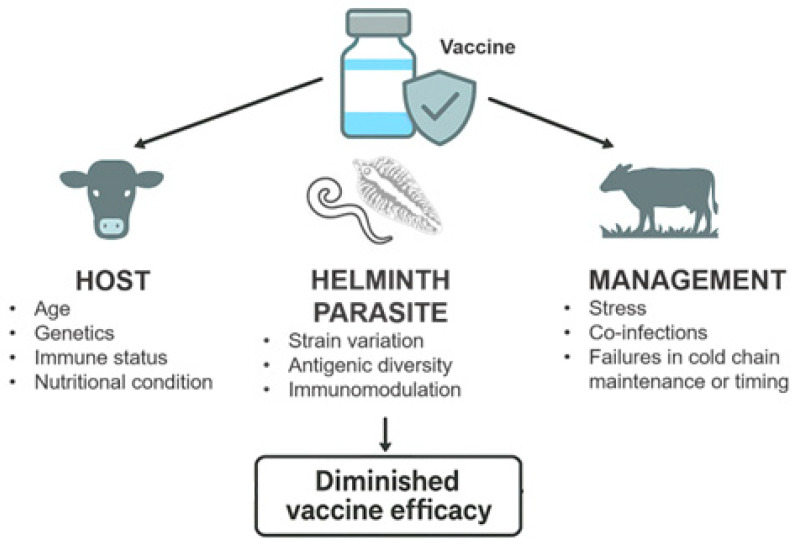
Factors influencing vaccine efficacy in livestock. Vaccine efficacy is determined by the interaction of host-, pathogen-, and management-related factors. Host factors include genetics, age, immune status, and nutritional condition. Pathogen-related factors include strain variation, antigenic diversity, and immunomodulatory capacity. Management-related factors comprise stress, concurrent infections, and failures in vaccine storage, handling, or timing. Together, these interacting factors can impair immune responses and lead to diminished vaccine efficacy under field conditions.

Consequently, animals exposed to persistent parasitic infections may exhibit attenuated responses to vaccination and reduced capacity to develop immunological memory [11]. Such infection-driven immunomodulation represents a major challenge for vaccine performance under field conditions, where animals are constantly exposed to endemic pathogens, and highlights the need for integrated disease management strategies that address co-infections alongside immunization programs (Figure 1).

## 5. Parasite Infection–Vaccine Interactions in Cattle

Environmental exposures, nutrition, and infection history continuously shape the immune system of cattle. Understanding how these infections interfere with vaccine-induced immunity is fundamental to improving disease control strategies and to adapting vaccination programs to diverse epidemiological settings.

Helminth infections are a major constraint to cattle health and productivity, leading to significant economic losses in livestock systems worldwide. Numerous studies have demonstrated that parasitic infections can modulate host immunity and influence responses to vaccination [12]. Two recent meta-analyses reported that helminth infections present at the time of vaccination were associated with poorer immunization outcomes [13,14]. Notably, chronic helminth infections appeared to exert stronger immunosuppressive effects than acute infections, suggesting that the duration and intensity of parasitic exposure play a critical role in shaping vaccine efficacy [13]. 

While most of this evidence comes from human studies and experimental models, data in cattle remain limited and sometimes inconsistent. The immune modulation induced by helminths, characterized by a Th2-biased cytokine profile, enhanced regulatory T cell activity, and increased production of IL-10 and TGF-β, can suppress Th1-driven responses required for protection against many viral and bacterial pathogens [15,16]. These alterations may impair antigen presentation, antibody class switching, and memory formation following vaccination.

However, most of these observations have been detected in the mouse model of infection with helminth parasites, which do not necessarily correlate with the ruminant system of cattle. Mice, in response to helminth infections, develop a strong and rapid Th2 immune response with high levels of IL-4, IL-5 and Il-13 [17]. On the other hand, helminth infections in bovines typically elicit a combined Th2/regulatory profile, with strong induction of IL-4, IL-13 and eosinophilia, but also robust upregulation of IL-10 and TGF-β, which suppress effector functions and limit tissue damage. Compared with mice, the bovine response is slower, heavily regulated, and rarely results in complete parasite clearance. Humans share certain regulatory features, but the bovine mucosal immune system shows a much stronger compartmentalization at the abomasum and small intestine. In addition, ruminants possess a distinctive and highly abundant population of γδ T cells expressing the workshop cluster 1 (WC1) coreceptor, which functions both as a pattern-recognition molecule and an activation modulator. These WC1^+^ γδ T cells represent up to 60% of circulating T cells in young calves and play a central role in early mucosal immunity during helminth infection [18,19]. Unlike γδ T cells in mice and humans, where they form a minor lymphocyte subset, bovine WC1^+^ cells mount rapid responses that shape downstream adaptive immunity. Last, WC1 molecules themselves act as multivalent scavenger receptor cysteine-rich (SRCR) pattern-recognition receptors capable of binding helminth-associated molecular patterns, enabling activation independently of classical MHC pathways [19]. This positions WC1^+^ γδ T cells as important early sensors of helminth invasion.

These species-specific differences are especially important when interpreting immunomodulation by helminths. For example, *Fasciola hepatica* and *Ostertagia ostertagi* both downregulate T-cell proliferation, antigen-presenting cell activity and antibody switching in cattle more profoundly than in murine models [20] Thus, extrapolating immunological findings from mice to ruminants requires caution, as the bovine immune system is uniquely adapted to chronic helminth exposure and displays regulatory pathways not represented in other species.

Understanding the extent and mechanisms of helminth-induced immunomodulation in ruminants is therefore essential to improve vaccination strategies in endemic areas and to develop integrated control programs that combine deworming and immunization for optimal herd protection.

### 5.1. Ostertagia ostertagi

One of the parasite infections that has been more extensively studied in ruminants is *Ostertagia ostertagi*, a gastrointestinal nematode that is one of the most significant parasites affecting cattle, particularly in temperate regions. The parasite develops in the abomasal glands, where larval invasion and emergence cause destruction of the gastric mucosa, leading to impaired acid secretion and disruption of the normal digestive process. Clinically, infected animals may present with diarrhea, weight loss, reduced feed efficiency, and in severe cases, submandibular edema and high morbidity. Even subclinical infections have significant consequences in production systems, as they reduce growth rates, milk yield, and overall productivity. Indeed, *O. ostertagi* infections in calves have been associated with a 15–30% reduction in average daily gain [21] and with 35–45% decreases in T-cell proliferation [22], both of which are markers likely to influence vaccine responsiveness. Therefore, *O. ostertagi* not only represents a significant animal health problem but also poses considerable economic losses to the cattle industry worldwide [23].

*O. ostertagi* employs multiple strategies to suppress host immunity. Its excretory/secretory products (ESPs) modulate innate immune responses by altering macrophage activation and toll-like receptor (TLR) signalling, downregulating costimulatory molecules (e.g., CD40), reducing proinflammatory cytokine production (IL-6, IL-1), and enhancing regulatory mediators such as IL-10 [24]. At the adaptive level, larval extracts inhibit proliferation of Concanavalin A-stimulated T lymphocytes, reduce IL-2 receptor expression, and suppress key T-cell cytokines including IL-2, IFN-γ, and TNF-α, while promoting IL-10 and TGF-β expression in mucosal tissues, thereby reinforcing a regulatory environment [25,26]. In addition, during *O. ostertagi* infection, WC1^+^ γδ T cells are rapidly recruited to the abomasal mucosa, where they respond to epithelial damage and helminth-derived ESPs [27]. Their activation leads to the production of IFN-γ in early infection, followed by IL-4 and IL-10 during chronic stages, contributing to the Th2/regulatory environment characteristic of bovine ostertagiosis [28]. Through these cytokines, WC1^+^ cells help balance inflammation and tissue repair, limiting epithelial injury while maintaining functionality of the gastric barrier. Recent transcriptomic analysis of abomasal mucosa from calves experimentally infected with *O. ostertagi* revealed a mixed Th1/Th2/Th17 response plus upregulation of T-cell exhaustion markers and a lack of expected epithelial alarmin induction, rather than a clear Th2-biased profile [27]. This indicates that nematode infection may induce a complex dysregulation of gastric immunity, potentially compromising the induction of protective vaccine responses.

Although several studies in humans, rodents, and pigs clearly show that nematode infections can affect vaccine responsiveness, evidence in ruminant species remains less conclusive [29]. Early research on *O. ostertagi* infections in cattle reported a transient, non-specific suppression of cellular immune responses during the initial stages of infection [30,31]. However, empirical field data in cattle remain scarce and inconsistent. In one study, serological evidence of *O. ostertagi* infection in dairy cows did not impair antibody response to rabies vaccination during the housing period [29]. Similarly, Schutz et al. found no significant effect of concomitant gastrointestinal parasite burden on serologic response to IBRV vaccination or challenge [32]. These findings highlight that the immunomodulatory impact of helminths may not always translate into reduced vaccine efficacy in cattle, and suggest that effects may depend on parasite species, burden, vaccine type, and management context, among other factors.

### 5.2. Fasciola hepatica

Fascioliasis is a food-borne trematodiasis caused by *Fasciola hepatica* and *Fasciola gigantica*, affecting millions of ruminants worldwide and resulting in annual economic losses exceeding US$3 billion. It is also a major zoonosis, classified by the World Health Organization as a neglected tropical disease, with about 17 million people infected and 180 million at risk [33]. In cattle, fascioliasis reduces productivity through liver damage, weight loss, and decreased milk yield. Control relies mainly on triclabendazole (TCBZ). Still, increasing reports of treatment failure and resistance in ruminants from Europe, South America, and Australia raise serious concerns for both animal health and potential human infections with resistant strains [34]. *F. hepatica* exerts a significant immunoregulatory effect on its hosts by inducing a predominantly regulatory immune response, characterized by an increase in regulatory T cells and immunosuppressive cytokines and reduced expression of type 1 cytokines like IL-2, IL-12, and IFN-γ, which are critical for effective cell-mediated immunity [20,35]. A recent transcriptomic profiling of PBMC from cattle experimentally infected with *F. hepatica* provides strong evidence that the parasite induces an early and sustained immunoregulatory phenotype rather than a classical pro-inflammatory response [36]. Importantly, the analysis revealed marked upregulation of genes associated with alternative activation and regulatory pathways, including IL-10 signaling, TGF-β-related transcripts, and inhibitory receptors, together with suppression of Th1/Th17-related genes and antigen-presentation pathways [36]. Notably, the PBMC transcriptional response was characterized by downregulation of key pathways involved in leukocyte activation, interferon signaling, and dendritic-cell maturation, consistent with a systemic shift toward immune dampening. Collectively, these findings demonstrate that *F. hepatica* establishes a broad immunomodulatory environment in cattle, skewing peripheral immunity toward regulatory/anti-inflammatory programs that may impair effective responses to concurrent infections and vaccines.

Although WC1^+^ γδ T cells are abundant in cattle and play important roles in mucosal and early immune responses, there is currently no direct evidence describing their behaviour during *F. hepatica* infection. Existing studies on bovine helminths indicate that γδ T cells can be modulated by parasite antigens, but their specific contribution to immunoregulation in fasciolosis remains unknown

Several studies have reported that *F. hepatica* can actively evade or modulate host immune responses, thereby reducing the efficacy of unrelated vaccines. Experimental infections have shown that *F. hepatica* impairs the development of protective immunity induced by bacterial and viral vaccines, including those targeting *Bordetella pertussis* [37]. In cattle, concurrent *F. hepatica* infection has been associated with significantly reduced antibody titers and lower IgG1 avidity following vaccination against FMDV [12]. These findings suggest that the Th2-polarized and regulatory environment induced by chronic fasciolosis suppresses the Th1-driven and neutralizing antibody responses required for optimal vaccine performance. Importantly, the immunomodulatory effects observed under experimental conditions may underestimate the impact occurring in the field, where animals are continuously exposed to reinfection. Field observations suggest that persistent or repeated exposure to *F. hepatica* exacerbates vaccine hyporesponsiveness, particularly in grazing systems with high infection pressure (Sala et al., unpublished data). This highlights the importance of considering endemic infection dynamics and reinfection cycles when interpreting vaccine efficacy and designing vaccination programs for field conditions.

Furthermore, these results indicate that *F. hepatica* affects the quality of humoral immune responses and emphasize the need to understand and mitigate the immunomodulatory impacts of the parasite, which are crucial for vaccine efficacy and for improving disease control strategies in cattle. 

However, the nature and context of infection influence the extent to which *F. hepatica* affects vaccine-induced immunity. In experimental models, *F. hepatica* infection did not significantly alter the antibody response elicited by a two-dose bovine respiratory syncytial virus (BRSV) vaccine, although a transient reduction in IgG1 levels was observed after priming and before the booster dose [38]. Similarly, *F. hepatica* infection did not substantially modify the humoral response to certain bacterial vaccines [38]. This variability likely reflects differences in the type of immune response required for protection and in the innate signalling pathways activated by each vaccine. Bacterial vaccines often rely on strong engagement of pattern recognition receptors (PRRs), such as TLRs, which can partially counteract helminth-induced immune regulation. In contrast, viral vaccines, particularly those that depend on robust Th1 or cytotoxic T-cell responses, may be more sensitive to the Th2 polarization and regulatory cytokine milieu associated with chronic helminth infections. More research is needed to identify which components of these vaccines actually counteract the regulatory effects exerted by *F. hepatica*. Overall, the impact of *F. hepatica* on vaccination appears to be context-dependent, determined by the nature of the pathogen, the vaccine platform, and the host immune environment at the time of immunization.

## 6. Possible Directions for Mitigating Helminth-Related Vaccine Interference

Helminth infections such as *F. hepatica* and *O. ostertagi* have been shown to impair vaccine-induced immune responses, emphasizing the need to integrate parasite management into vaccination programs. Effective parasite control can enhance vaccine performance by reducing helminth-driven immunosuppression, but it must also balance economic feasibility and the risk of accelerating anthelmintic resistance.

Treatment of *F. hepatica* relies mainly on TCBZ, which is effective against immature and adult stages, although resistance is increasingly common; other drugs such as closantel, nitroxynil, or clorsulon target mostly adult flukes [39]. For *O. ostertagi*, macrocyclic lactones, benzimidazoles, and levamisole remain the primary anthelmintic options, though resistance is also emerging [40]. *A priori*, blanket pre-vaccination deworming could theoretically minimize parasite-mediated immunosuppression, yet this approach is not sustainable due to cost, resistance development, and environmental considerations.

Although *F. hepatica* infection clearly induces systemic immunomodulation in cattle [36], evidence that anthelmintic treatment consistently reverses this dysregulation prior to vaccination is currently lacking in bovines. Experimental models in rodents indicate that deworming alone may be insufficient to restore vaccine efficacy in some settings [41,42], but well-designed longitudinal trials in cattle are needed to test whether similar persistence occurs under field conditions. These findings suggest that while reducing parasite burden is desirable, the expectation that pre-vaccination deworming will fully restore vaccine responsiveness may be unrealistic in some contexts.

## 7. Diagnostic Approaches to Support Targeted Deworming

Accurate diagnosis is fundamental to identifying infected animals before vaccination and to guiding appropriate treatment. For *F. hepatica*, fecal egg counts (FECs) are widely used but detect only patent infections from 10–12 weeks post-infection, resulting in substantial false negatives in early or low-intensity infections. Intermittent egg shedding and sample contamination, particularly in extensive systems, may further compromise sensitivity and specificity [34]. Coproantigen ELISAs, which detect parasite antigens in feces, can identify active infections as early as 6–8 weeks post-infection and are more sensitive for monitoring treatment success [43,44]. However, antigen degradation during transport can reduce sensitivity, and antigen persistence following treatment may contribute to false positives and misinterpretation of therapeutic success. Antibody ELISAs in serum or milk can reveal exposure within 2–4 weeks after infection and are particularly useful for herd-level surveillance. Nevertheless, antibody persistence long after parasite clearance often leads to false positives, limiting their utility for individual-level decision-making prior to vaccination. Access to milk testing facilitates implementation in dairy systems, whereas serum sampling in extensive beef operations poses logistical challenges [45,46]. Abattoir liver inspection continues to provide valuable prevalence data but lacks timeliness for on-farm decision-making.

In recent years, molecular assays have substantially enhanced the sensitivity and specificity of *F. hepatica* diagnosis. Semi-nested and SYBR Green real-time PCRs enable early and precise detection of parasite DNA in feces, outperforming conventional serology in terms of specificity and turnaround time [47]. Multiplex formats such as duplex PCR allow simultaneous identification of *F. hepatica* and other trematodes like *Clonorchis sinensis* [48]. More recently, isothermal amplification-based methods such as loop-mediated isothermal amplification (LAMP) and recombinase polymerase amplification (RPA) combined with CRISPR/Cas12a detection have shown great potential for field application, providing rapid, visual, and equipment-free diagnosis of copro-DNA [48,49]. These molecular tools expand the diagnostic window to earlier stages of infection and may complement serological and coproantigen tests in integrated surveillance systems. However, fecal inhibitors frequently generate false negatives, while detection of DNA from non-viable parasites can cause false positives. Although promising for earlier diagnosis, these assays typically require specialized laboratory facilities, trained personnel, and higher per-sample costs, limiting their real-world utility, and the high per-sample cost restricts routine application prior to vaccination.

For *O. ostertagi*, FECs remain the standard herd-level tool, although the lack of species specificity limits precision [50]. Coproculture followed by larval differentiation allows species identification but requires 10–14 days [51]. Serum pepsinogen activity is a sensitive indicator of abomasal damage caused by *O. ostertagi* larvae and is particularly valuable for detecting subclinical infections in calves [52]. However, it lacks species specificity, as elevated pepsinogen levels may result from nutritional or metabolic stress rather than parasitism. Thus, false positives are common, and interpretation requires context-specific thresholds that are not universally validated. Antibody ELISAs on serum or milk support epidemiological studies and herd monitoring but cannot differentiate between past and active infections [53].

Across all these methods, production system constraints markedly influence diagnostic accuracy and feasibility. Notably, extensive grazing systems face major barriers to repeated individual sampling, controlled sample handling, and laboratory access, increasing the likelihood of diagnostic error. In intensive or dairy systems, laboratory-based assays are more feasible but still limited by cost, turnaround time, and challenges in differentiating active from past infections.

Overall, no single diagnostic tool currently provides high sensitivity, specificity, affordability, and logistical feasibility across diverse production environments. A combined diagnostic approach including coproantigen ELISA and FEC for *F. hepatica*, and FEC with serum pepsinogen or larval culture for *O. ostertagi*, should provide the most reliable foundation for targeted deworming prior to vaccination. There remains, however, a gap in tools capable of detecting early infections with high sensitivity and specificity under field conditions. Integrating advanced molecular assays into vaccination planning could help reduce helminth-related immune suppression and support more sustainable, evidence-based anthelmintic use.

## 8. Implementation and Integrated Management

Although helminth infections can alter immune pathways relevant to vaccine responsiveness, the number of controlled studies directly evaluating the effect of prior helminth infection on vaccine efficacy in cattle is still limited. Therefore, recommendations regarding parasite management before vaccination must be interpreted within this context of partial and heterogeneous evidence. Rather than constituting a universally validated requirement, parasite control should be considered a potentially beneficial and biologically plausible strategy to reduce immunomodulatory pressure, particularly in production systems where high infection burdens are common.

Despite practical challenges and variable farmer compliance, incorporating evidence-based parasite control into vaccination programs is crucial. Demonstrating both economic and immunological benefits can motivate adoption. High-risk groups, such as first-grazing-season calves with high *Ostertagia* burdens or late-gestation cows in fluke-endemic areas where the freshwater snail of the family *Lumnaeidae* is present, may be particularly susceptible to helminth-associated immune modulation. In these contexts, targeted anthelmintic treatment administered two to three weeks before key vaccinations has the potential to reduce parasite-mediated immunosuppression, although the magnitude of this effect likely varies across herds and management conditions. Diagnostics, such as FECs, coproantigen ELISAs, or herd-level serology, can identify infected animals and provide objective evidence to support treatment decisions, improving farmer compliance and reducing unnecessary drug use.

Integration of management practices is essential for long-term control. Pasture management, including rotation and avoidance of overgrazing or wet areas that favor snail intermediate hosts, reduces reinfection pressure, while appropriate nutrition enhances vaccine and parasite resilience [26,44,54]. Monitoring herd performance metrics (weight gain, milk production, fertility, morbidity) allows assessment of treatment efficacy and reinforces the link between parasite control and productivity [55]. In addition, in the case of fasciolosis, elimination or reduction of the intermediate snail host can complement chemotherapy and pasture management, decreasing parasite transmission and thereby reducing parasite-mediated immunosuppression, although practical implementation is most feasible in targeted high-risk areas and must be integrated with other control measures [56,57].

**Figure 2:**
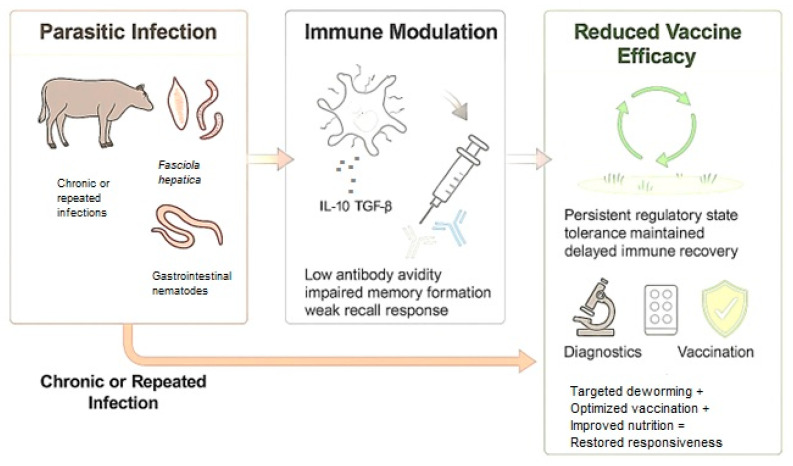
Parasite-Immunity-Vaccine Interaction Cycle in Cattle. Chronic or repeated parasitic infections, including *F. hepatica* and gastrointestinal nematodes, modulate host immune responses by promoting regulatory cytokines such as IL-10 and TGF-β. This immune modulation leads to reduced antibody avidity, impaired memory cell formation, and weakened recall responses, ultimately diminishing vaccine efficacy. Integrated control strategies such as combining targeted deworming, optimized vaccination timing, and improved nutrition, can restore immune responsiveness and enhance protective outcomes in cattle under endemic infection pressure.

Finally, education and herd-health planning are critical. Farmers should be made aware that subclinical parasitism represents “hidden losses” that compromise productivity and vaccine efficacy [58]. Framing parasite control supported by a comprehensive vaccine optimization strategy, and collaborating with cooperatives or extension services to provide training, diagnostics, and demonstration herds, can foster sustainable adoption of diagnostic-based, targeted control approaches that enhance vaccine performance and livestock health (Figure 2).

## 9. Conclusions

Helminths and other parasites modulate both innate and adaptive immune responses through altered antigen presentation, cytokine dysregulation, and the induction of regulatory or exhausted immune phenotypes. These changes can impair B-cell activation, reduce antibody class switching and avidity, and limit the formation and maintenance of memory cells, ultimately compromising long-term vaccine-induced protection [15].

An important consideration is that most experimental models of parasite–vaccine interactions rely on controlled, single infections, which only partially reproduce the immunological landscape observed in the field. Under natural grazing conditions, cattle are frequently exposed to continuous or repeated infections that sustain a regulatory and anti-inflammatory immune environment. This persistent stimulation reinforces immune tolerance, disrupts memory cell persistence, and delays the restoration of vaccine responsiveness even after deworming. Consequently, vaccine efficacy measured under experimental conditions may overestimate protection compared with the complex reinfection scenarios faced in endemic areas [13]. Post-vaccination monitoring using validated correlates of protection is paramount to assess the success of a vaccination strategy under field conditions. Therefore, the impact of helminth infections on vaccine efficacy in cattle should be further assessed under field conditions.

Addressing these challenges requires integrated health management strategies that combine parasite control with immunization programs adapted to endemic settings. Strategic, diagnostic-based anthelmintic use, complemented by biosecurity and nutritional management, can improve vaccine outcomes by reducing parasite-driven immunoregulation before immunization. The development of next-generation vaccines, including subunit, recombinant, mRNA, and antiparasitic formulations, represents a long-term goal to reduce dependence on chemotherapy and enhance host resilience. Although antiparasitic vaccines remain scarce, there are encouraging indications that their number will increase in the coming decade [59].

Although most available anthelmintics remain effective and affordable, the global rise in resistance, concerns over drug residues, and the lack of novel compounds highlight the urgency of developing complementary immunological approaches [60]. Achieving this goal requires a multidisciplinary framework linking parasitology, immunology, and livestock management [61]. Collaborative efforts among research institutions, industry, and veterinary services under private–public partnerships will be essential to translate immunological insights into field-applicable solutions. Through such coordinated strategies, it will be possible to improve vaccine performance, mitigate the immunosuppressive effects of parasitic infections, and promote the sustainability and productivity of livestock systems.

## Data Availability

No new data were created or analyzed in this study. Date sharing is not applicable to this article.

## Abbreviations

The following abbreviations are used in this manuscript:

BHV-1	bovine herpesvirus-1
BVDV	bovine viral diarrhea virus
CSF	classical swine fever
FMD	Foot-and-Mouth Disease
FMDV	Foot-and-Mouth Disease virus
GMP	Good Manufacturing Practices
IL	Interleukin
PRRSV	respiratory syndrome virus
TD	T-cell dependent
TI	T-cell independent
